# Preparation of aminated porous polyacrylonitrile nanofibers as adsorbent for methyl orange removal

**DOI:** 10.1039/d2ra00780k

**Published:** 2022-05-19

**Authors:** Qinghua Wu, Xionghui Ling, Weigeng Huang, Xianhua Zeng, Longfei Fan, Junyu Lin, Wenhui Yu, Jiaen Yao, Wu Wen

**Affiliations:** School of Textile Materials and Engineering, Wuyi University 22# Dongcheng Village Jiangmen Guangdong Province People's Republic of China andytsang32@163.com; Guangdong Provincial Key Laboratory of Industrial Surfactant, Institute of Chemical Engineering, Guangdong Academy of Sciences China

## Abstract

In this study, porous electrospinning polyacrylonitrile nanofiber (PPAN) surface functionalization with amine groups is studied for methyl orange (MO) dye removal from aqueous solution. A series of adsorption experiments were carried out to investigate the influence of initial solution pH value, contact time, initial solution concentration, and adsorption temperature on the adsorption performance. The experimental results showed that the removal of MO on these PPAN-PEI and PPAN-TEPA nanofibrous mats was a pH-dependent process with the maximum adsorption capacity at the initial solution pH of 3, and that the PPAN-PEI and PPAN-TEPA nanofibrous mats could be regenerated successfully after 4 recycling processes. The adsorption equilibrium data were all fitted well to the Langmuir isotherm equation, with maximum adsorption capacity of 1414.52 mg g^−1^ and 1221.09 mg g^−1^ for PPAN-PEI and PPAN-TEPA, respectively. The kinetic study indicated that the adsorption of MO could be well fitted by the pseudo-second-order equation and Weber–Morris model. Thermodynamic parameters such as free energy, enthalpy, and entropy of adsorption of the MO were also evaluated, and the results showed that the adsorption was a spontaneous exothermic adsorption process.

## Abstract 2

Amino functionalized porous polyacrylonitrile electrospun nanofibers were fabricated, which have good adsorption performance for MO in an acidic environment.

## Graphical abstract

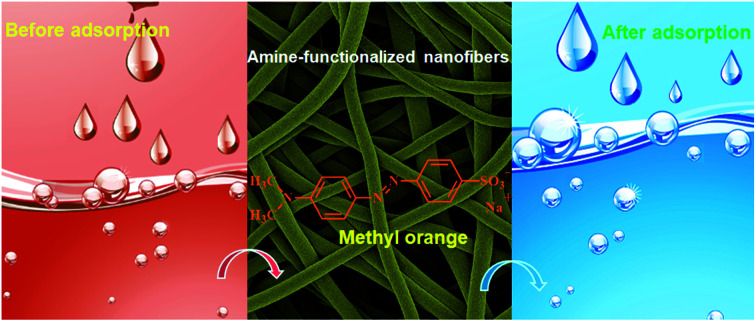

## 1 Introduction

Synthetic dyes, which are widely used in industrial fields such as leather, plastics, printing, or textiles, are considered a major source of pollution in wastewater because of their potential carcinogenicity, teratogenicity, and hard-to-degrade properties. Therefore, the elimination of dyes from wastewater has become an urgent environmental problem to be solved. Currently, various methods have been established including membrane filtration,^1^ photocatalysis,^2^ biodegradation,^3^ electrochemical oxidation,^4,5^ and adsorption^6,7^ for the treatment of synthetic dyes-contained wastewater. Among these methods, adsorption has attracted extensive attention due to its simple adsorption/desorption operation, high efficiency, and economical features. Many different kinds of adsorbents like activated carbon,^8–10^ zeolite,^11^ graphene oxide,^12^ carbon nanotubes,^13^ and MOFs^14,15^ have been employed to remove the dyes. However, utilization of these powdered adsorbents is limited in practical application the filtration steps along with sample loss and generation of secondary pollution, high cost of production, low selectivity. Therefore, it is important to prepare a better adsorption performance adsorbent to overcome those shortcomings.

Nanofibers produced by the electrospinning method can be used as one type of ideal dye absorption material in the wastewater due to their special structure of nanoscale, large specific surface area, excellent flexibility, and recovery. For example, a series of different average fiber diameter nanofibrous membranes with pure chitosan were prepared nanofibrous membranes using electrospinning technology by Li *et al.*^16^ The maximum adsorption capacity of 1377 mg g^−1^ was achieved for acid blue-113 adsorption by the chitosan nanofibrous membrane with average fiber diameter of 86 nm. In addition, good regeneration of pure chitosan nanofibrous membranes was observed after four cycles. Xu *et al.* fabricate the nanofibrous membrane by using PES as the matrix through electrospinning technology. And the obtained nanofibrous membrane named UFAM showed excellent performances of recyclability for nearly 80% after 10 desorption–resorption cycles, selective adsorption, and smart filtration–separation for cationic and anionic dyes. In particular, the as-prepared UFAM was found to be exceptionally efficient in the removal of methylene blue (MB) dye from aqueous solution.^17^ Moreover, a large variety of polymers can also be used to produce nanofibrous mats by electrospinning for water treatment, including polylactic acid (PLA),^18^ polyethylene terephthalate (PET),^19^ polyethylene oxide (PEO),^20^ polyvinyl alcohol (PVA),^21,22^ polystyrene (PS),^23,24^ polyvinylidene fluoride (PVDF),^24,25^ and polyacrylonitrile (PAN).^26–28^ PAN was chosen as the matrix in this study due to its chemical stability in the sewage environment, superior mechanical performance, low cost, as well as abundant nitrile groups (C≡N) that can participate in targeted chemical reactions to anchor specific adsorption groups.^29,30^ Nevertheless, PAN nanofibers are usually difficult to adsorb MO effectively because of the lack of interaction force. So, it is necessary to introduce the functional groups like amino, which own high affinity to form electrostatic attraction with anionic dyes like MO by protonated of amino, and the adsorption capacity can increase dramatically.^31,32^

In this work, we have chemically modified the surface of the PPAN electrospinning the nanofibers matrix by using polyethyleneimine (PEI) and tetraethylenepentamine (TEPA) to generate a positive charge on the nanofibers' surface. PEI and TEPA were employed as the functional compounds because of their abundant primary amine (–NH_2_), secondary amine (–NH–) groups. Then, subsequently, investigations into the adsorption behaviors of the amino-functionalized PPAN nanofibers towards MO removal, adsorption kinetics, thermodynamics as well as reusability studies have been carried out and the maximum adsorption capacity obtained has been compared with different reported nanoadsorbents.

## 2 Materials and methods

### 2.1 Materials

Polyacrylonitrile (PAN, *M*_w_ = 85 000), poly(vinyl pyrrolidone) (PVP, *M*_w_ = 1 300 000), *N*,*N*-dimethylformamide (DMF), polyethyleneimine (PEI, *M*_w_ = 10 000, 99%), tetraethylenepentamine (TEPA, 95%), ethylene glycol, methyl orange (MO, 98%), sodium hydroxide (NaOH) were all bought from Macklin Biochemical Co., Ltd. HCl (36–38 wt%) was provided by Guangzhou Chemical Reagent Factory. All chemicals were used as received. Deionized (DI) water produced by a Water Purification System (UPT-11-20T) was used throughout this work.

### 2.2 Preparation of porous PAN nanofibers

PAN/PVP NFMs were fabricated by the electrospinning technique as follows: briefly, 8 wt% of PAN powder was added into DMF slowly with vigorous magnetic stirring for 1 h. Subsequently, PVP powder (PAN/PVP ratio = 8/10) was dissolved in the above PAN/DMF solution for 12 h at 40 °C. Then, the as-prepared homogeneous PAN/PVP solution was transferred into a plastic syringe (10 mL), and a tip-to-collector distance of 20 cm was maintained. A high voltage of 30 kV and a feed rate of 1.5 mL h^−1^ were applied. The obtained bicomponent nanofibers were dried at 70 °C to remove the remaining solvent of DMF. Afterward, the fabricated PAN/PVP NFMs were transferred to a Teflon stainless-steel autoclave with DI water and hydrothermally treated at 100 °C for 8 h to remove PVP. After being cooled to room temperature naturally, the liquor was decanted, and the obtained white membranes were washed several times with DI water to remove the residual extracted PVP. After drying the water-treated nanofibers at 70 °C for 8 h, the porous PAN nanofibers, named PPAN were obtained.

### 2.3 Preparation of amino-functionalized porous electrospun PAN nanofibers

**Scheme 1:**
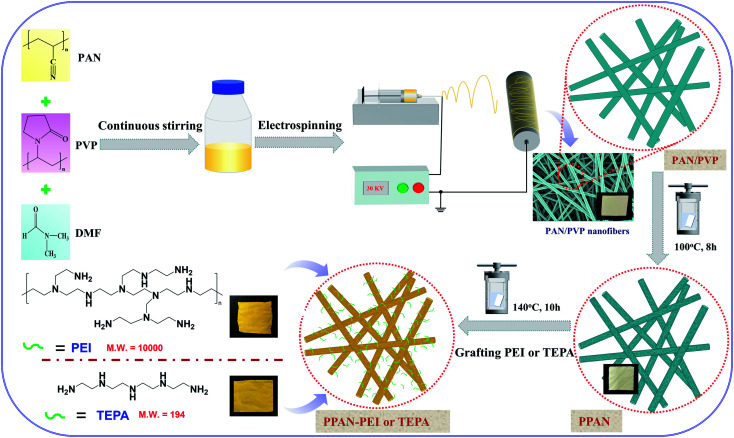
Schematic illustration of the synthesis of amine-grafted porous electrospun PAN nanofibers.

The amino-grafted PPAN nanofibers process was described briefly as follows. First, a certain amount of electrospun porous PAN fibers and 60 mL PEI or TEPA and ethylene glycol mixture (10 wt% PEI or TEPA) were sonicated for 15 minutes at room temperature before added into a 100 mL Teflon stainless-steel autoclave, and then placed into a vacuum oven at 140 °C (PEI grafting temperature) or 120 °C (TEPA grafting temperature) for different times to obtain different grafting degrees of nanofibers. After the reaction, the PEI or TEPA grafted electrospun porous PAN fibers (PPAN-PEI or PPAN-TEPA) were washed with deionized water and ethanol several times, and finally dried in a vacuum oven at 60 °C overnight. The grafting degree of PEI or TEPA onto the PPAN was determined by the weight gain of PPAN-PEI or PPAN-TEPA, which was determined using eqn (1):1
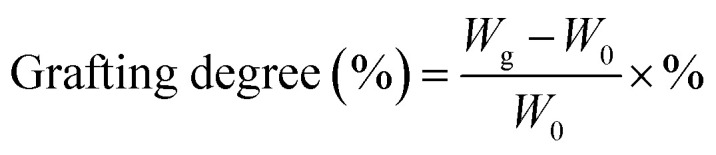
where *W*_0_ is the weight of the raw PPAN, and *W*_g_ is the weight of the PEI or TEPA grafted PPAN-PEI or PPAN-TEPA. And the whole fabrication process is presented in Scheme 1.

### 2.4 Characterization

The surface and cross-sectional morphology of the nanofibers were observed by field emission scanning electron microscopy (SEM, Sigma 300-ZEISS). The mean diameter of the nanofibers was calculated from measuring the different parts of the fibers at 100 different fibers for mean diameter using the free software package, Image-J. FT-IR spectra were obtained on a Fourier transform infrared spectrometer (FT-IR, THERMO FISHER) from 4000 to 400 cm^−1^.

### 2.5 Batch adsorption procedure

All the batch adsorption experiments were performed on a model SHA-B shaker with a shaking speed of 120 rpm. The MO solution was prepared by dissolving in deionized water with the concentration of 1000 mg L^−1^ and was then diluted to the required various concentration before use. The initial pH values of the solution were adjusted with 0.1 M HCl or 0.1 M NaOH, and the pH values were measured using a pH meter. The concentration of MO was determined by METASH UV-600 spectrophotometer at 464 nm. The adsorption capacity (*q*) and removal efficiency (RE%) of MO onto PPAN-PEI and PPAN-TEPA were calculated from the following equations:2
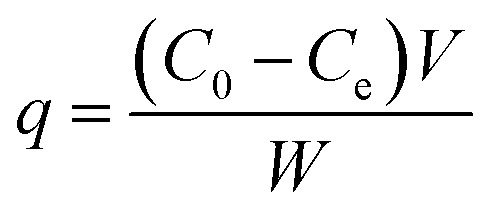
3
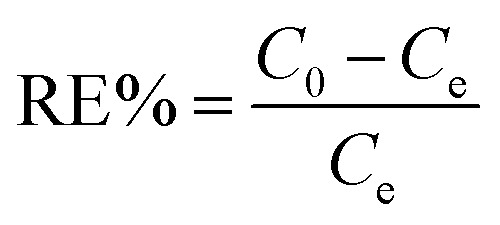
where *q* is the adsorbed amount (mg g^−1^), *W* is the weight of the fiber (mg), *V* is the volume of solution (mL), and *C*_0_ and *C*_e_ are the initial and equilibrium concentrations (mg L^−1^) of MO in the test solution, respectively.

The initial pH of the adsorption solution was adjusted to values in the range of 3–8 for MO to investigate pH effects. The adsorption kinetic was conducted with an initial concentration of 25 mg L^−1^ at 30 °C. Adsorption isotherms were conducted with initial concentrations ranging from 2 to 150 mg L^−1^. The dose of adsorbents was about 4 mg for 30 mL test solution in 50 mL Erlenmeyer flasks. To investigate the recyclability of the amino-functionalized nanofibers, the saturated MO-adsorbed nanofibers were washed thoroughly with 0.1 M NaOH. Then washing the electrospun membrane to neutral with deionized water after desorption equilibrium. The samples were reused in adsorption experiments repeating 4 times with an initial concentration of 25 mg L^−1^ at 30 °C.

## 3 Results and discussion

### 3.1 Characterization of the prepared nanofibers

**Fig. 1:**
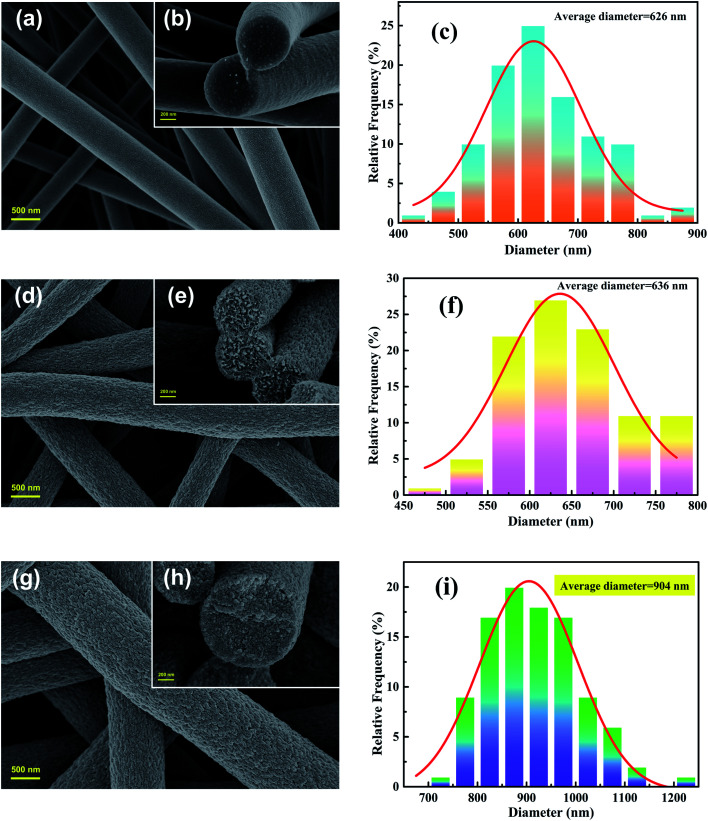
FESEM images (left: superficial images; right: cross-sectional images) and diameter distributions of nanofibers: PAN/PVP (a–c), PPAN (d–f), and PPAN-PEI (g–i).

The images of Fig. 1 indicate that the overall experimental processes of the obtained nanofibers all keep good fibrous morphology, suggesting that the water-soluble PVP and grafting reaction don't destroy the fibrous structures. From the Fig. 1(a and b) observations, the obvious cracks were observed because the two polymers of PVP and PAN are partially miscible in DMF. As shown in Fig. 1(d and e), the porous PPAN nanofibers were obtained successfully by solution method based on the principle that PVP can easily dissolve into the water. Moreover, numerous pores were observed both on the surface and inner of the fibers, indicating that the nanofibers were porous throughout.^28^ In addition, the average diameters of the PAN/PVP and PPAN (Fig. 1(c and f)) are very closed, which demonstrated the PPAN fibers can remain intact structure after the removal of PVP at high temperature. However, the fiber diameter distributions show that the average fiber diameter of PPAN-PEI increases significantly compared with PPAN nanofibers (Fig. 1(i and f)), indicating that the PEI has been loaded successfully. Fortunately, as presented in Fig. 1(g and h) there still were a certain number of pores among the amine-functional nanofibers, which could improve the probability of contact between the amino and MO, thus enhancing the adsorption capacity. The above results indicate the feasibility of our experimental approach.

**Fig. 2:**
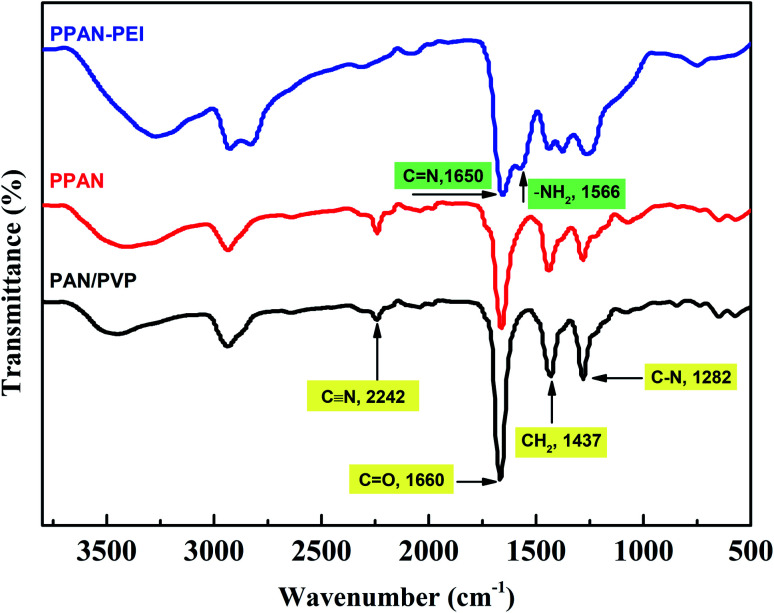
The FT-IR spectra of the PAN/PVP, PPAN, and PPAN-PEI.

The FT-IR spectra (Fig. 2) were used to study the composition of the obtained fibers and the reaction processes. The spectra of PAN/PVP and PPAN display a band at 2242 cm^−1^, which belongs to C≡N stretching vibration, indicating the presence of PAN.^33,34^ The band located at 1660 cm^−1^ is attributed to the C=O stretching vibration; the band located at 1437 cm^−1^ was assigned to the CH_2_ bending vibration, and the band located at 1282 cm^−1^ is attributed to the stretching vibration of C–N.^33^ These three characteristic peaks proved the existence of PVP. It is found in the spectra of PPAN that the strength of these characteristic absorption peaks of PVP is weak, revealing that part of the PVP has been removed and some of the PVP remained in the PPAN nanofibers. The reason for this might be that PVP and PAN were partially miscible, and the entangled polymer chains formed semi-interpenetrating polymer networks resulting in the fact that PVP was embedded and cannot be removed even by hot water.^35,36^ The spectra indicate the intensity of the peak at 2242 cm^−1^ (C≡N) disappeared after PEI grafting, and two peaks at 1650 cm^−1^ and 1566 cm^−1^ appear respectively, indicating the existence of C=N and N–H groups through grafting.^34,37^ The results confirm that the amino group and cyano group can react with each other in ethylene glycol solution.^38^

### 3.2 Adsorption performance of amino-functionalized nanofibers

Through the above-mentioned characterization and discussion, we have demonstrated the successful preparation of flexible and robust amino-functionalized nanofibers, which are beneficial for the practical adsorption applications. The adsorption performance of PPAN-PEI and PPAN-TEPA toward MO was also investigated and shall be discussed in the following section.

#### 3.2.1 Effect of initial pH

**Fig. 3:**
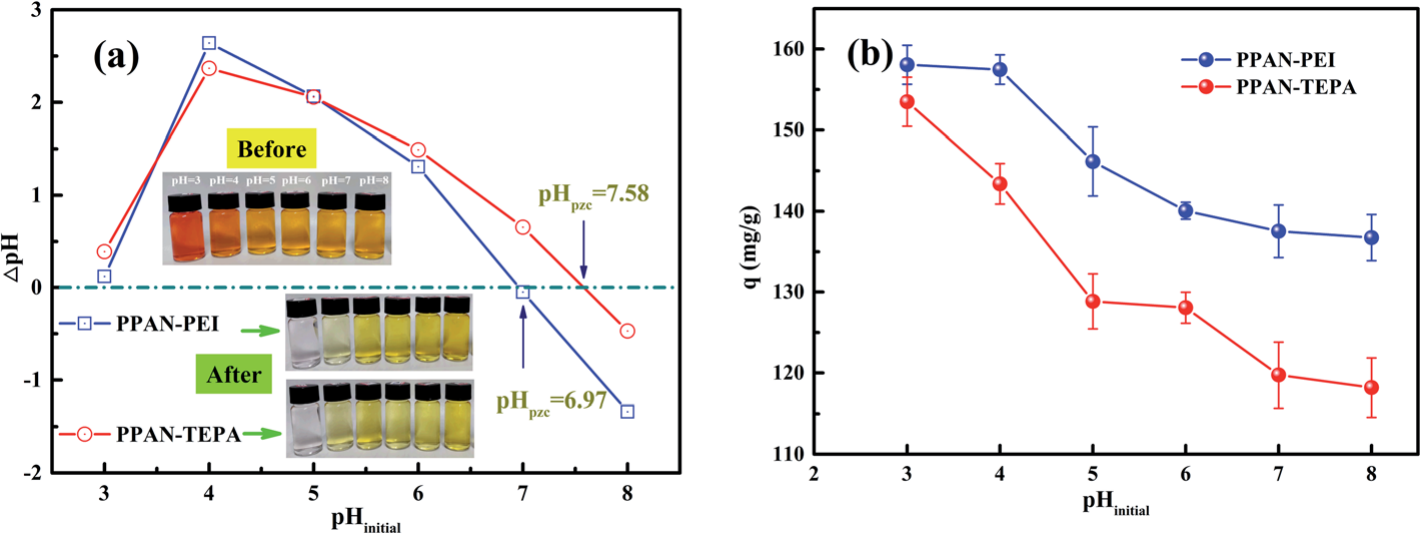
(a) The ΔpH (pH_final_ − pH_initial_) against pH_initial_ plots for PPAN-PEI and PPAN-TEPA nanofibers, and (b) the adsorption capacity of PPAN-PEI and PPAN-TEPA nanofibers at different pH_initial_.

**Scheme 2:**
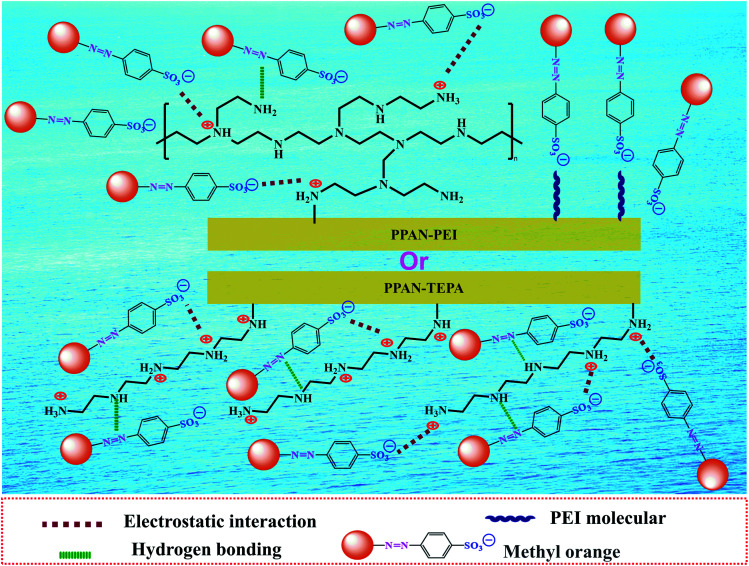
Schematic illustration of the adsorption mechanism of amino-functionalized nanofibers toward MO.

The pH value is an important factor affecting adsorption for dye. Firstly, to better understand the adsorption process of surface charge of the amino-functionalized nanofibers, the point of zero charge (pH_pzc_) for PPAN-PEI and PPAN-TEPA nanofibers were measured. As the results present in Fig. 3(a), the pH_pzc_ values are 7.58 and 6.97 for PPAN-PEI and PPAN-TEPA, respectively. The high pH_pzc_ value implies the cationic acid character for both adsorbents, which favors the adsorption of the anionic pollution.^39^ And a positive charge of PPAN-PEI and PPAN-TEPA can be obtained at a pH environment below the pH_pzc_, which prefers the adsorption of the anionic pollutant such as MO. The effect of the initial pH value on the adsorption of MO onto PPAN-PEI and PPAN-TEPA also was investigated at different pH values ranging from 3.0 to 8.0 and the results were shown in Fig. 3(b). It is evident that the maximum MO removal is observed at pH 3, and gradual decreases in the MO removal can be observed for both adsorbents by increasing the pH value towards the basic environment. This was attributed to that the surface of PPAN-PEI and PPAN-TEPA would be positively charged for the protonation of surface amino groups when the pH value decreased below pH_pzc_.^40–42^ On the other hand, the sulfonate group in the molecular structure of the MO could be converted in aqueous medium into active negative sulfonate group (Scheme 2). Consequently, a strong electrostatic attraction between PPAN-PEI or PPAN-TEPA and MO thus increases dyes adsorption.

Moreover, the decrease in the adsorption capacity and color removal of MO with increasing pH value can be attributed to the competition between anionic dyes and excess hydroxyl ions in the solution. Therefore, this optimal pH 3 value was employed for all subsequent adsorption experiments.

#### 3.2.2 Effect of the grafting degree of the adsorbent on its MO adsorption

**Fig. 4:**
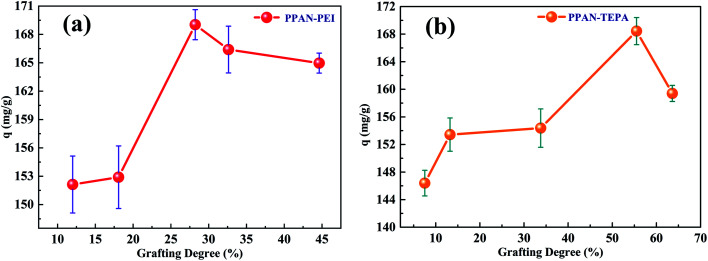
Adsorption capacity of PPAN-PEI (a) and PPAN-TEPA (b) with different grafting degrees.

The influence of the grafting degree of PPAN-PEI and PPAN-TEPA nanofibers on the adsorption capacity was examined, respectively. As shown in Fig. 4, after a certain value of grafting degree is reached, the adsorption capacity is no longer significantly increased. Increasing the grafting degree of PEI or TEPA, the chains are entangled more easily for PEI, and the steric hindrance increases after MO is adsorbed. In summary, a higher grafting degree may have resulted in a stronger mass transfer resistance of MO into the inner part of the grafting layer. Consequently, not all the amine groups in the inner part could contact with MO; this led to the adsorption capacity does not increase as the grafting degree increased.

#### 3.2.3 Comparative adsorption

**Fig. 5:**
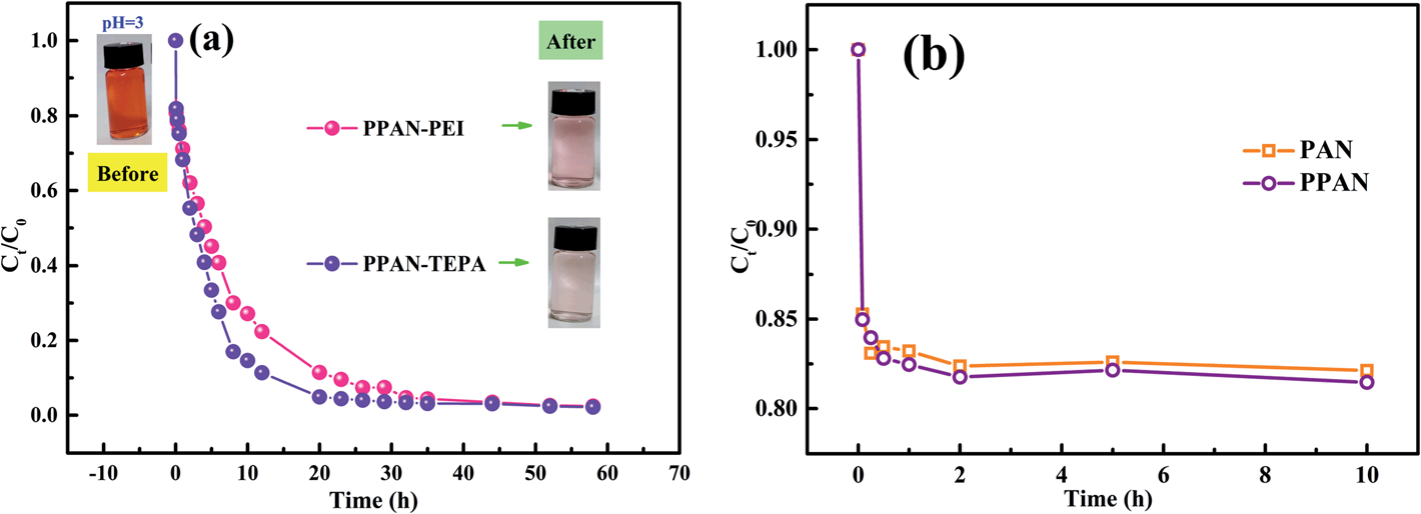
Time dependence of relevant nanofibers adsorption rate for MO capture.

To explore the change of adsorption capacity after grafted PEI and TEPA, a comparative adsorption test of related adsorbents was studied. As shown in Fig. 5(a), the experimental groups treated by PPAN-PEI and PPAN-TEPA are significantly higher removal efficiencies of MO than PAN/PVP and PPAN. After 23 h adsorption, the removal efficiencies for PPAN-PEI and PPAN-TEPA are 90.44% and 95.59%, respectively. However, as shown in Fig. 5(b) the removal efficiencies of PAN and PPAN can reach approximately 15% in 1 h and remain unchanged, which may be attributed to physical adsorption for MO onto PAN and PPAN. Similar results, such as the adsorption rate of raw nanofibers is higher than the modified nanofibers but the adsorption capacity is lower, have also been reported in other studies.^26,32^ The results revealed that the amines could effectively improve the adsorption capacity of PPAN toward MO. Moreover, the removal efficiency of PPAN-TEPA was slightly higher than PPAN-PEI, which may be because of the greater steric hindrance of the PEI molecular.

#### 3.2.4 Adsorption isotherms

Adsorption isotherms are fundamental for evaluating the adsorption capacity of an adsorbent. Langmuir and Freundlich models were used for the non-linear fitting of the experimental data.^51^ The models can be expressed by the following nonlinear equations:

Langmuir model eqn (4):4
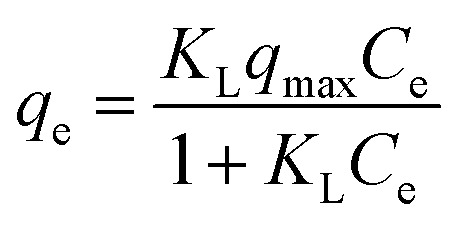
Freundlich model eqn (5):5
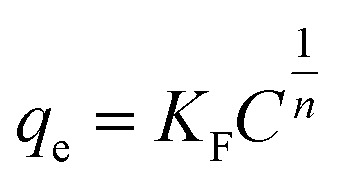
where *C*_e_ (mg L^−1^) is the concentration in the solution at equilibrium; *q*_max_ is the maximum adsorption capacity of the adsorbent; *K*_L_ (L mg^−1^) is Langmuir constant; *K*_F_ is Freundlich constant, *n* is the intensity of the adsorbent.

**Fig. 6:**
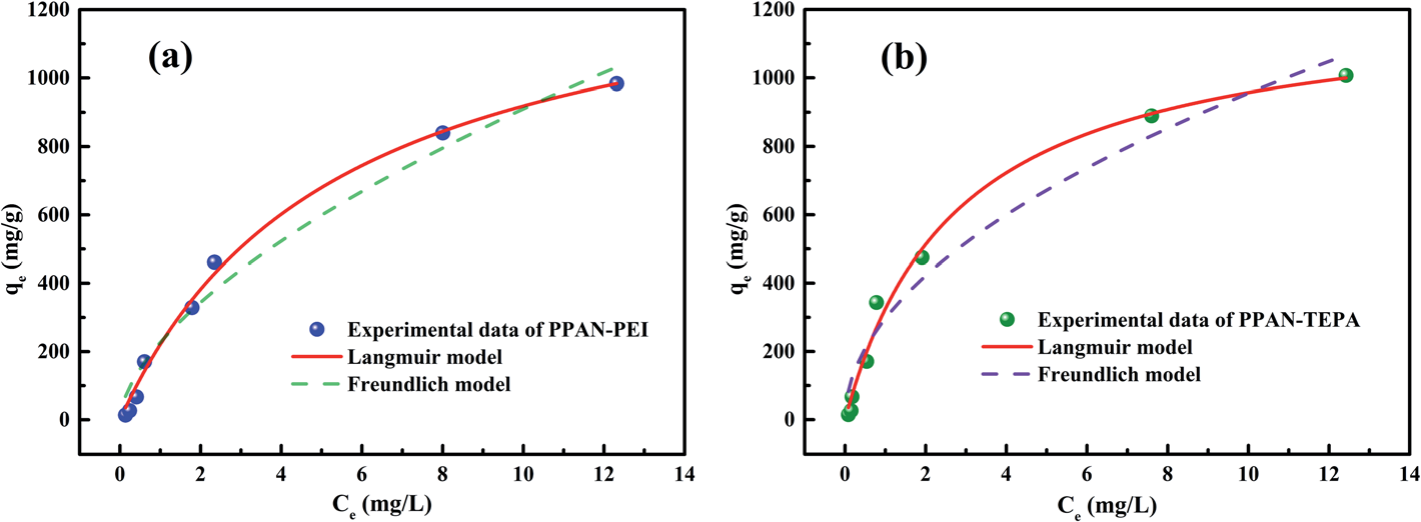
The fitting plots of Langmuir and Freundlich parameters of PPAN-PEI (a) and PPAN-TEPA (b), respectively.

Fig. 6 shows the non-linear fitting line of the adsorption isotherm model, and the fitting parameters obtained by the fitting results were listed in Table 1. The adsorption processes of MO by PPAN-PEI and PPAN-TEPA are more matched with the Langmuir model (Fig. 6(a and b)). Compared with the Freundlich model, the correlation coefficient (*R*^2^) of the Langmuir model is closer to 1 (Table 1), which suggests that the adsorption took place at specific homogeneous sites within the adsorbent forming monolayer coverage of MO at the surface of the PPAN-PEI or PPAN-TEPA nanofibers. Besides, the obtained 1/*n* values below 1 which support the removal MO processes are based on chemical adsorption for PPAN-PEI and PPAN-TEPA.^52^ Furthermore, the maximum adsorption capacities of PPAN-PEI and PPAN-TEPA for MO are 1414.52 mg g^−1^ and 1221.09 mg g^−1^ at 30 °C, respectively. The maximum amount of MO in this work is compared to other adsorbents recently reported in the literature as shown in Table 2. The adsorption capacity of prepared composite fibers for MO is comparatively higher than some other adsorbents.

**Table 1:** Langmuir and Freundlich parameters of PPAN-PEI and PPAN-TEPA toward MO adsorption

Adsorbent	Langmuir model			Freundlich model		
	*q*_max_	*K*_L_	*R* ^2^	1/*n*	*K*_F_	*R* ^2^
PPAN-PEI	1414.52	0.1852	0.9940	0.6036	226.65	0.9725
PPAN-TEPA	1221.09	0.3628	0.9903	0.5080	296.60	0.9615

**Table 2:** Comparison of the maximum adsorption capacity (*q*_max_) of MO on various adsorbents

Adsorbent	*q*_max_ (mg g^−1^)	Ref.
Amine-modified PIM-1 fibrous membrane	312.5	43
The chitosan/polyvinyl alcohol/zeolite electrospun composite nanofibrous membrane	153	44
3D rGO/ZIF-67 aerogel	426.3	45
The protonated amine-modified hydrochar (PAMH)	909.09	31
PEI modified spent tea leaves	62.11	46
ACNTs	253.26	47
ZIF-67@LDH	1029.59	48
Amine rich porous PAN nanofibers	254	32
Lignin-derived zeolite templated carbon materials	514	49
Multi-functional polyethersulfone nanofibrous membranes	909.8	50
PPAN-PEI	1414.52	This work
PPAN-TEPA	1221.09	This work

**Fig. 7:**
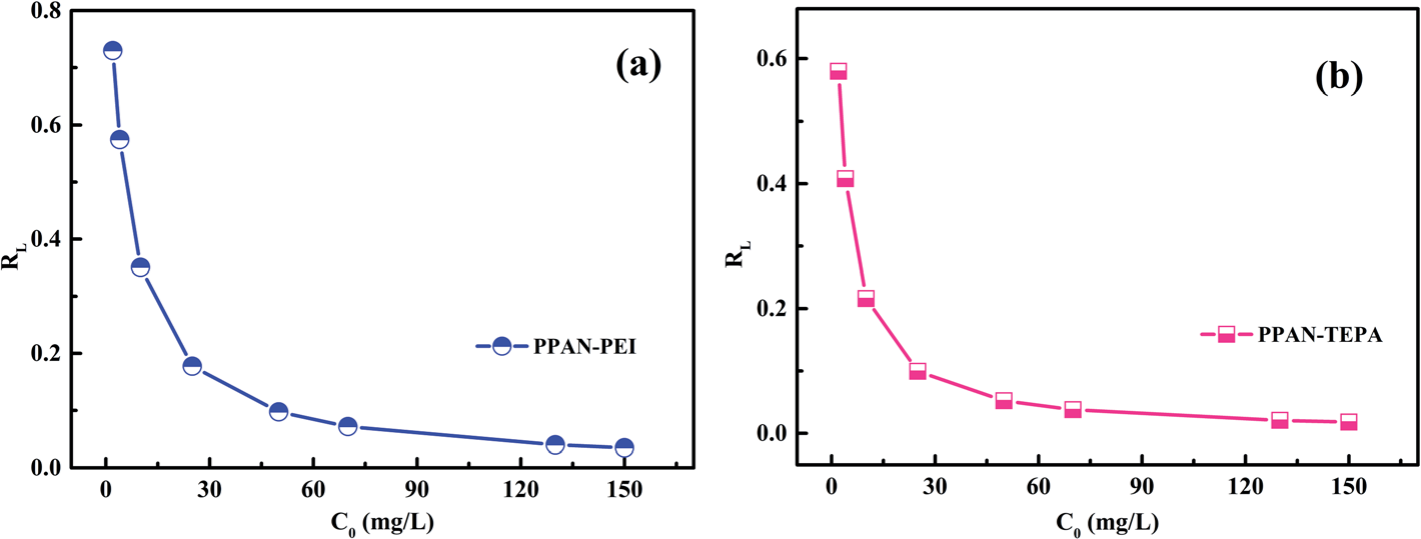
*R*_L_ for the adsorption of MO onto PPAN-PEI (a) and PPAN-TEPA (b), respectively.

The dimensionless separation factor *R*_L_ obtained by using Langmuir parameters shown in Table 1 can be calculated as follows:6
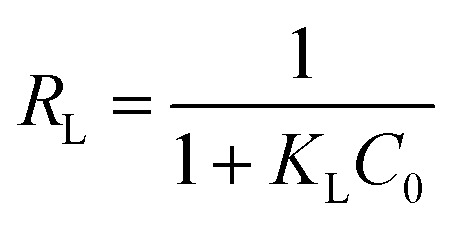
where *K*_L_ (L mg^−1^) was the adsorption equilibrium constant of the Langmuir isotherm model. *C*_0_ (mg L^−1^) was the original concentration of MO dye. This factor is a criterion of the tendency between adsorbate and adsorbent indicating the isotherms be either unfavorable (*R*_L_ > 1), linear (*R*_L_ = 1), favorable (0 < *R*_L_ < 1) or irreversible (*R*_L_ = 0).^53^ As shown in Fig. 7, the *R*_L_ values of PPAN-PEI and PPAN-TEPA are in the range of 0–1, suggesting that the adsorption is a favorable uptake process, particularly for PPAN-TEPA.

#### 3.2.5 Adsorption kinetics

**Fig. 8:**
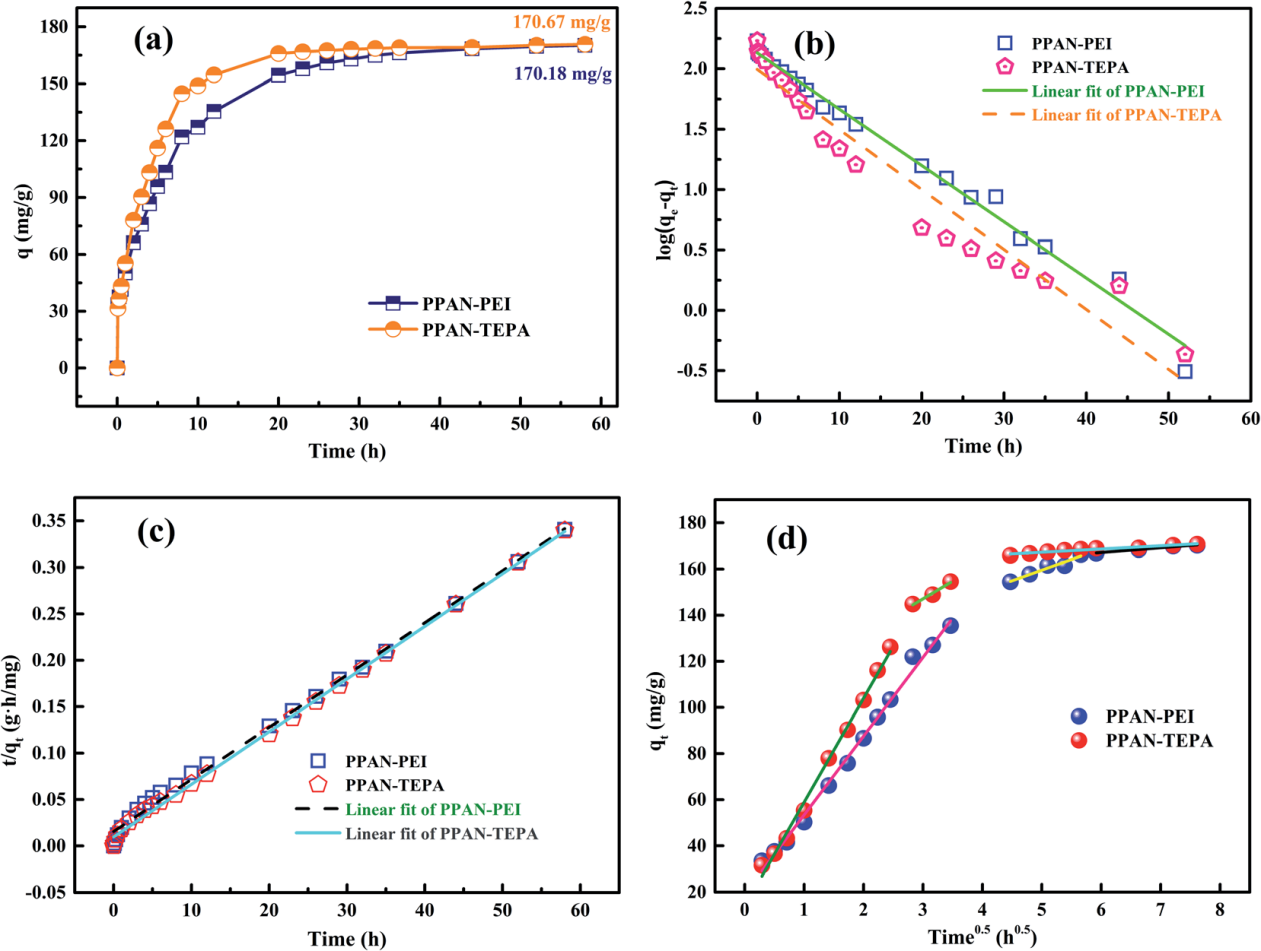
Adsorption kinetics curves of MO onto PPAN-PEI and PPAN-TEPA (a), their pseudo-first-order kinetic plots (b) and pseudo-second-order kinetic plots (c), and Weber–Morris model (d) graphs.

The effect of contact time on MO sorption by PPAN-PEI and PPAN-TEPA was displayed in Fig. 8(a). It can be observed that the sorption rate sharply increased within 8 h due to a large number of available adsorption sites on both PPAN-PEI and PPAN-TEPA surfaces. And then MO molecules came into the nanofibers because most of the amines have been consumed, leading to a slow adsorption rate until the adsorption equilibrium.

To further explore the adsorption process, the adsorption kinetic curve of MO adsorption by PPAN-PEI and PPAN-TEPA was measured by employing two kinds of widely-used kinetic models: the pseudo-first-order and pseudo-second-order rate equations.^53^ Their linear forms of the equation can be given as:7
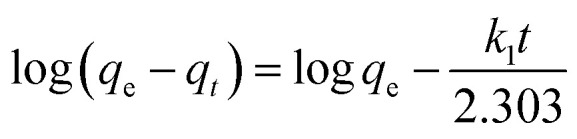
8
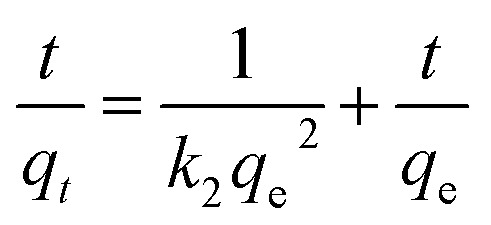
where *q*_e_ (mg g^−1^) and *q*_*t*_ (mg g^−1^) are the amounts of MO adsorbed at equilibrium and at time *t* (h), respectively; *k*_1_ (h^−1^) and *k*_2_ (g mg^−1^ h^−1^) are the pseudo-first-order and pseudo-second-order rate constants of adsorption, respectively.

The rate constants (*k*_1_ and *k*_2_) and corresponding linear regression correlation coefficient values (*R*^2^) for both models were calculated using the intercept and slope of the two plots shown in Fig. 8(b and c) and summarized in Table 3. It can be seen that the values of *R*^2^ from the pseudo-second-order equation are higher than those from the pseudo-first-order equation, and the calculated *q*_e_ values (*q*_e,cal_) from the pseudo-second-order equation and experimental *q*_e_ values (*q*_e,exp_) match very well each other, showing the pseudo-second-order equation is more suited to depict the MO sorption kinetic and the sorption process is controlled by chemisorption for PPAN-PEI and PPAN-TEPA toward MO adsorption.

**Table 3:** Kinetic parameters for MO adsorption by PPAN-PEI and PPAN-TEPA, respectively

Adsorbent	Experimental *q*_exp_	Pseudo-first-order model			Pseudo-second-order model		
		*q*_e,cal_ (mg g^−1^)	*k*_1_ (h^−1^)	*R* ^2^	*q*_e,cal_ (mg g^−1^)	*k*_2_ (g mg^−1^ h^−1^)	*R* ^2^
PPAN-PEI	**170.18**	135.39	1.85 × 10^−3^	0.9878	**177.94**	2.03 × 10^−3^	0.9953
PPAN-TEPA	**170.67**	98.53	1.97 × 10^−3^	0.9459	**176.37**	3.31 × 10^−3^	0.9984

The intraparticle distribution model named Weber-Morris model^54^ was also employed to further study the MO diffusion mechanism during the adsorption process for PPAN-PEI and PPAN-TEPA. Its linear form is shown as follows:

9 *q*_*t*_ = *k*_d_*t*^0.5^ + *C*

where *k*_d_ is the intra-particle distribution rate constant and *C* is the thickness of the boundary layer. As displayed in Fig. 8(d), the adsorption processes can be divided into three stages. In the first stage, MO molecules move toward the exterior surface of nanofibers and the adsorption rate is faster. In the second stage, the fitted curves exhibit a significantly lower slope in virtue of the decrease of available active adsorption sites and the increased steric hindrance caused by MO molecular. The third stage is the final equilibrium step resulting from very low MO concentration remaining in the solution. Furthermore, none of the lines pass through the origin, suggesting that intraparticle distribution is not the only rate-controlling step in the whole adsorption process for both absorbents (Table 4).

**Table 4:** Parameters of Weber and Morris model of MO onto PPAN-PEI and PPAN-TEPA nanofibers

Weber-Morris model						
Adsorbent	*K*_d1_ (mg g^−1^ h^−0.5^)	*R* ^2^	*K*_d2_ (mg g^−1^ h^−0.5^)	*R* ^2^	*K*_d3_ (mg g^−1^ h^−0.5^)	*R* ^2^
PPAN-PEI	31.14	0.9934	9.23	0.9268	2.06	0.9694
PPAN-TEPA	45.07	0.9943	15.32	0.9687	1.40	0.9351

#### 3.2.6 Adsorption thermodynamic

**Fig. 9:**
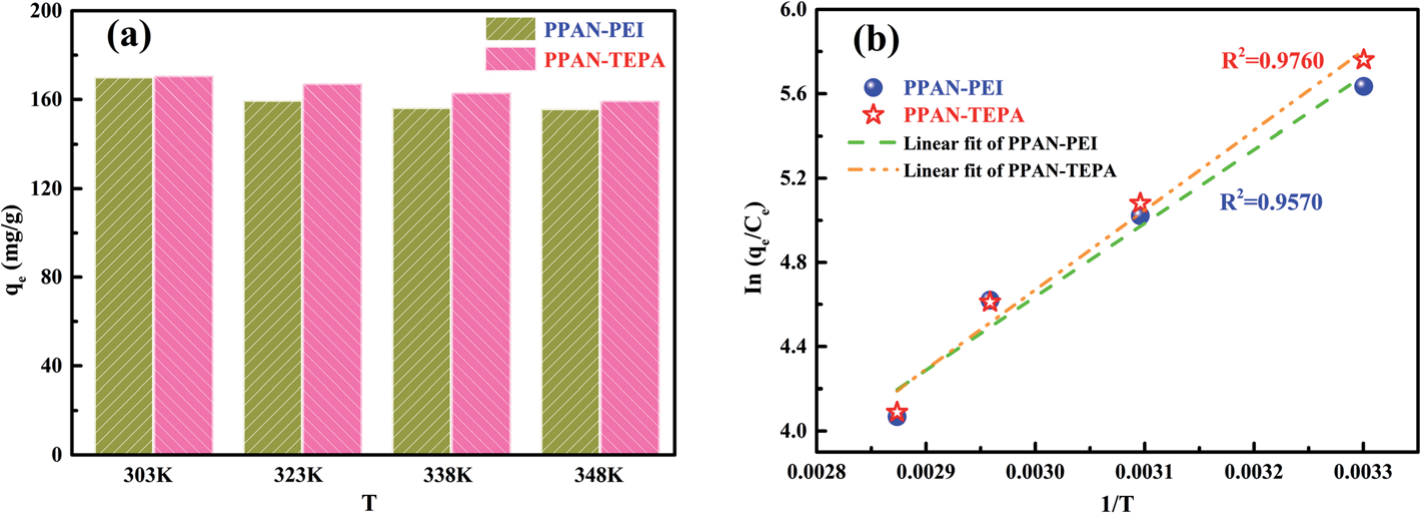
(a) Adsorption capacities of PPAN-PEI and PPAN-TEPA for MO at different temperature, respectively. (b) Plots of ln(*q*_e_/*C*_e_) against 1/*T* for the adsorption of MO onto PPAN-PEI and PPAN-TEPA, respectively.

Since the thermodynamic parameter of the adsorption process strongly depends on the temperature, the effect of temperature on the adsorption performance of MO was also studied. As shown in Fig. 9(a), the adsorption capacity of MO for both PPAN-PEI and PPAN-TEPA adsorbents decreases with the increase of temperature, indicating that the adsorption process is an exothermic process. To further investigate the spontaneity of the adsorption process, the adsorption data were analyzed by the famous Van't Hoff equation^55^ as follows:10
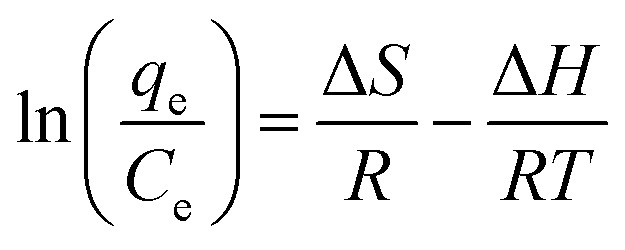
where *q*_e_ and *C*_e_ have the same definitions aforementioned. *R* is the universal gas constant (8.314 J mol^−1^ K^−1^), *T* (K) is the absolute temperature, and Δ*H* (kJ mol^−1^) and Δ*S* (J mol^−1^ K^−1^) are the change of enthalpy and entropy, respectively. The changes of Gibbs free energy Δ*G* (kJ mol^−1^) can be calculated from the following equation:11
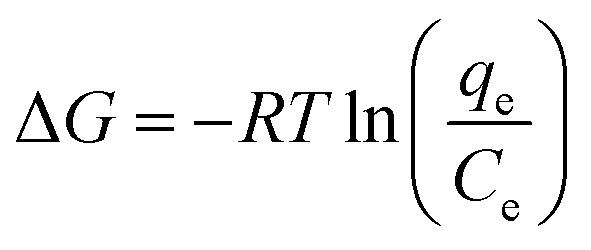
Plotting ln(*q*_e_/*C*_e_) against 1/*T* presents straight lines with high correlation coefficient (*R*^2^) values of 0.9570 and 0.9760 for PPAN-PEI and PPAN-TEPA (Fig. 9(b)), respectively, indicating that the Van't Hoff equation fits well with the adsorption data. The values of thermodynamic parameters for PPAN-PEI and PPAN-TEPA were similar and calculated based on the fitting results and were listed in Table 5. The negative values of Δ*H* and Δ*S* reveal that the MO adsorption process by the PPAN-PEI and PPAN-TEPA are exothermic in nature and is a randomness decrease at the solid–solution interface. Generally, adsorption can be classified into physical adsorption and chemical adsorption according to the absolute value of △H: the heat of adsorption is range from 2.1 to 20.9 kJ mol^−1^, and 80 to 200 kJ mol^−1^ represents physisorption and chemisorption, respectively.^56^ The absolute values (29.05 kJ mol^−1^ and 31.45 kJ mol^−1^) indicated the adsorption of MO onto both adsorbents *via* chemisorption (the formation of a strong ionic bond) and physisorption (weaker van der Waals forces) simultaneously. Furthermore, the negative value of Δ*G* suggests that the adsorption process between PPAN-PEI or PPAN-TEPA and MO is spontaneous and no energy input is needed, as well as is negatively affected by temperature increases.

**Table 5:** The thermodynamic parameters for the adsorption of MO onto PPAN-PEI and PPAN-TEPA adsorbents

Adsorbent	Δ*H* (kJ mol^−1^)	Δ*S* (J mol^−1^ K^−1^)	Δ*G* (kJ mol^−1^)			
			303 K	323 K	338 K	348 K
PPAN-PEI	−29.05	−48.60	−14.3	−13.4	−12.6	−12.1
PPAN-TEPA	−31.45	−55.54	−14.6	−13.5	−12.7	−12.1

#### 3.2.7 Reusability evaluation

**Fig. 10:**
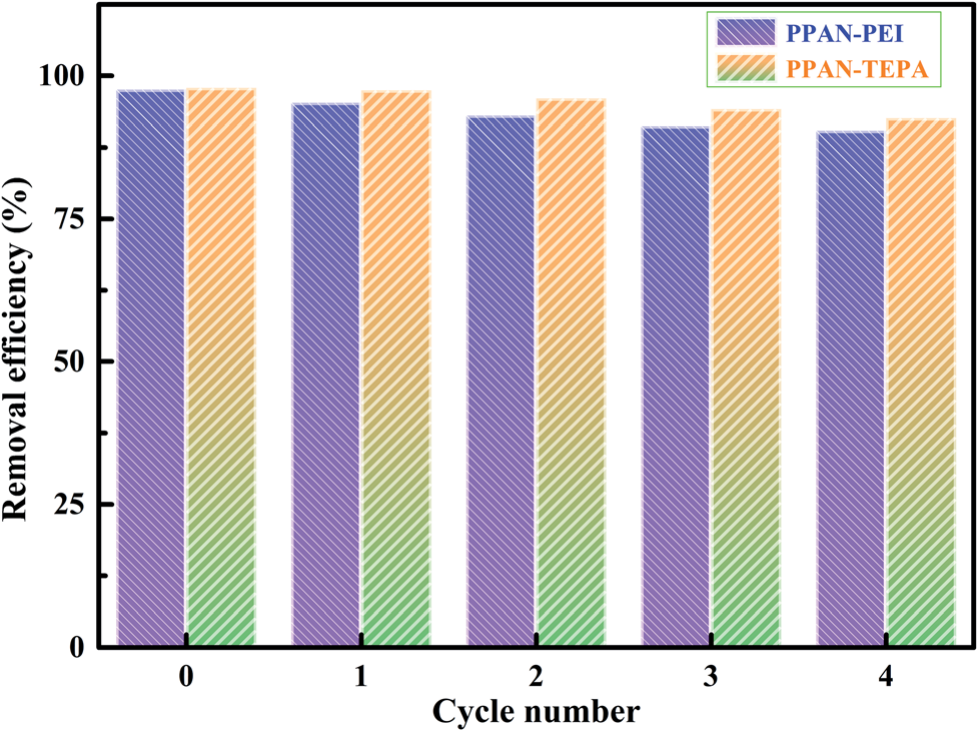
The recycling performances of PPAN-PEI and PPAN-TEPA.

The recycling and regeneration of the absorbent material are essential for its economic viability and actual application. To test the suitability and stability of the adsorbent, adsorption–desorption experiments have been carried out using 25 mg L^−1^ MO dye solution. Here, 0.1 M NaOH was chosen as an eluent according to the results of Fig. 10 to desorb MO from the MO-loaded PPAN-PEI and PPAN-TEPA. After desorption, PPAN-PEI and PPAN-TEPA were reused for adsorption. In each cycle, the adsorbents were repeatedly washed with deionized water after each desorption to eliminate the excess of the base until the pH of the solution was neutral. The results showed in Fig. 10 that the removal efficiency of MO can keep about 90.41% and 92.55% after four cycles for PPAN-PEI and PPAN-TEPA, respectively. This indicated that PPAN-PEI and PPAN-TEPA could be repeatedly used for the removal of MO from aqueous solution.

**Fig. 11:**
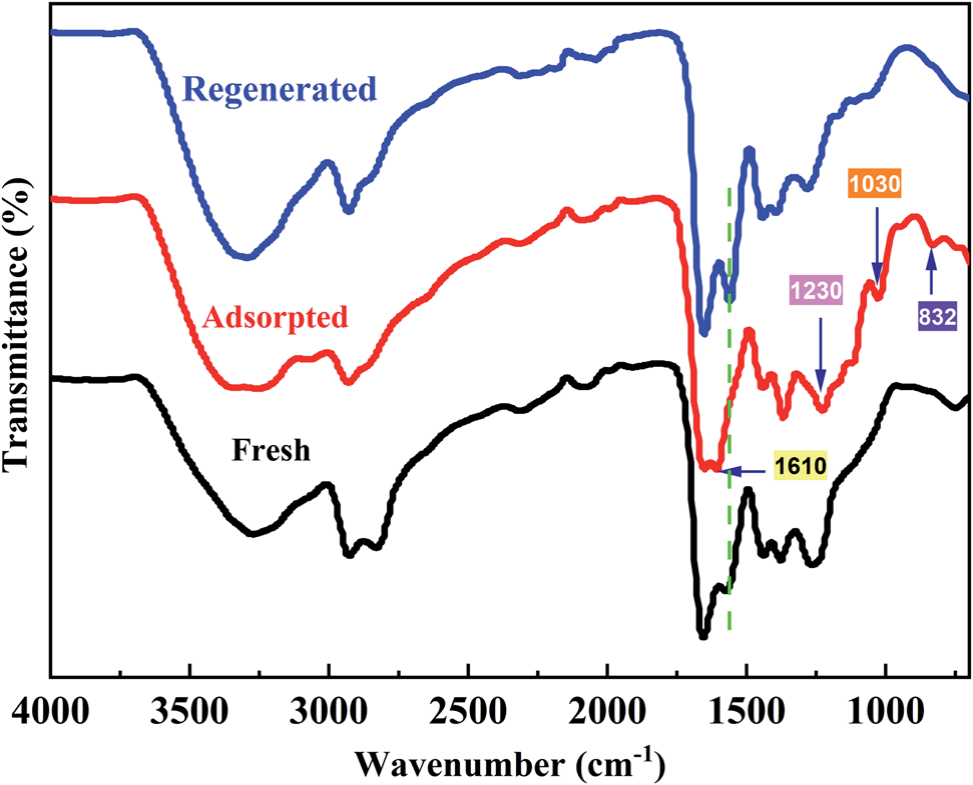
FT-IR spectra of the fresh, adsorbed, and regenerated PPAN-PEI.

The FT-IR spectra of the fresh PPAN-PEI, the PPAN-PEI after adsorption of MO and the regenerated fiber are illustrated in Fig. 11. Compared with the spectrum of fresh PPAN-PEI, the presence of new bands appearing at 1230, 1030, and 832 cm^−1^,^57,58^ which correspond to the characteristic peaks of MO, confirmed the successful adsorption of MO onto PPAN-PEI. Meanwhile, the characteristic absorption band belonging to N–H for –NH_2_ has shifted from 1566 cm^−1^ to 1610 cm^−1^ and is observed after MO adsorption under acid conditions, suggesting that the PEI of PPAN-PEI plays an important role in the electrostatic interaction ascribed to the protonation, which makes the surface of the adsorbent positively charged. The spectra of fresh and regenerated PPAN-PEI showed similar IR peaks and intensity, indicating the grafted PEI stayed intact during the MO adsorption–desorption cycles.

## 4 Conclusions

In this study, branched polyethylenimine and tetraethylenepentamine functionalized porous polyacrylonitrile electrospun fiber adsorbents (PPAN-PEI and PPAN-TEPA) were fabricated by the combination of electrospinning and grafting methods. PEI or TEPA played an important role in improved adsorption ability, which endowed the nanofibers high adsorption capacity toward MO with good recyclability, as well as high removal efficiencies (>97%) in both batch adsorption as the initial concentration of MO was 25 mg L^−1^ at 30 °C. More importantly, the removal efficiency kept above 90% after five adsorption–desorption cycles. In the batch adsorption, the adsorption behavior followed the pseudo-second-order order model and Langmuir isotherm. The obtained maximum adsorption capacity of PPAN-PEI was 1414.52 mg g^−1^ which was higher than some adsorbents. The thermodynamics study revealed that the adsorption process was spontaneous and exothermal. Based on the obtained results, PPAN-PEI and PPAN-TEPA electrospun fibers could be effective candidates in the wastewater treatment field.

## Conflicts of interest

There are no conflicts to declare.

## Supplementary Material
